# Distinguished Faculty Award Lectures

**DOI:** 10.1093/geroni/igaa057.3209

**Published:** 2020-12-16

**Authors:** Cynthia Hancock

## Abstract

The Distinguished Faculty Award lecture will feature an address by2020 recipient Mary W. Carter, PhD. The Distinguished Faculty Award acknowledges individuals whose teaching stands out as exemplary, innovative, or influential-- or any combination thereof.

